# The millipede family Polydesmidae in Taiwan, with descriptions of five new species (Polydesmida, Diplopoda)

**DOI:** 10.3897/zookeys.93.1167

**Published:** 2011-04-29

**Authors:** Sergei I. Golovatch, Elena V. Mikhaljova, Hsueh-Wen Chang

**Affiliations:** 1 Institute for Problems of Ecology and Evolution, Russian Academy of Sciences, Leninsky pr. 33, Moscow 119071, Russia; 2 Institute of Biology and Soil Science, Far Eastern Branch, Russian Academy of Sciences, Prospekt Stoletiya Vladivostoka 159, Vladivostok 690022, Russia; 3 Department of Biological Sciences, National Sun Yat-Sen University, 70 Lien-Hai Rd., Kaohsiung 804, Taiwan, ROC

**Keywords:** millipede, Polydesmidae, taxonomy, new species, new synonymy, key, Taiwan

## Abstract

Polydesmidae are represented in Taiwan by seven species in two genera. Neither of the genera is endemic to Taiwan, but six of the species are, including five new: *Nipponesmus minor* **sp. n.**, *Epanerchodus bispinosus* **sp. n.**, *Epanerchodus curtigonopus* **sp. n.**, *Epanerchodus flagellifer* **sp. n.** and *Epanerchodus pinguis* **sp. n.** In addition, the diagnosis of the hitherto enigmatic genus *Nipponesmus* Chamberlin & Wang, 1953 is refined vis-à-vis the especially similar, Central Asian, Siberian and Eastern European genus *Schizoturanius* Verhoeff, 1931, chiefly based on new material of the type-species *Nipponesmus shirinensis* Chamberlin & Wang, 1953; this species is adequately redescribed and represents still another Taiwanese endemic. A key to all three currently known species of *Nipponesmus* Chamberlin & Wang, 1953 is given. The highly speciose Central to East Asian genus *Epanerchodus* Attems, 1901 is represented in Taiwan by five species, all keyed, including *Epanerchodus orientalis* Attems, 1901, which is long known to be highly variable in Japan and found particularly polymorphous and apparently allochthonous in Taiwan. The following synonymy is formalized: *Epanerchodus orientalis orientalis* Attems, 1901 = *Epanerchodus orientalis takakuwai* Verhoeff, 1913, **syn. n.** The genus *Usbekodesmus* Lohmander, 1932 is formally synonymized with *Epanerchodus* Attems, 1901, **syn. n.**, resulting in the following new formal transfers: *Epanerchodus redikorzevi* (Lohmander, 1932), *Epanerchodus swatensis* (Golovatch, 1991), *Epanerchodus varius* (Geoffroy & Golovatch, 2004), *Epanerchodus anachoretus* (Golovatch, 1986), *Epanerchodus buddhis* (Golovatch, 1986), *Epanerchodus occultus* (Golovatch, 1986), *Epanerchodus sacer* (Golovatch, 1987), *Epanerchodus theocraticus* (Golovatch, 1990) and *Epanerchodus theosophicus* (Golovatch, 1986), all **comb. n.** ex *Usbekodesmus*. The distributions of all seven species of Polydesmidae occurring in Taiwan are mapped and discussed.

## Introduction

The millipede fauna of Taiwan is still quite poorly explored (Korsós 2004), with fewer than 70 species currently reported from this large, subtropical to tropical island. Many more species may be expected to occur there, to judge from the recent increase in the list of Taiwanese Glomerida from one species to five (Golovatch et al. 2010). This statement is easy to reconfirm with the large family Polydesmidae, order Polydesmida, taken as another example. Taiwan has hitherto been known to support only two polydesmid species: *Epanerchodus orientalis* Attems, 1901 and *Nipponesmus shirinensis* Chamberlin & Wang, 1953, both from a few localities only (see review: Korsós 2004).

Prompted by the abundant new material collected during the last couple of decades all over the island, below we offer a revision of the Taiwanese Polydesmidae. Already seven species appear to be involved, representing two genera. These new samples also allow for the diagnosis of one genus to be refined, and the synonymy of another one formalized.

## Material and methods

Material serving as the basis for the present contribution was preserved in 75% alcohol and is currently shared between the collections of the National Museum of Natural Science, Taichung, Taiwan (NMNS), Taiwan Forest Research Institute, Taipei, Taiwan (TFRI), Department of Biological Sciences,National Sun Yat-Sen University, Kaohsiung, Taiwan (NSYSU), Zoological Museum, State University of Moscow, Russia (ZMUM), Natural History Museum of Denmark, University of Copenhagen, Denmark (ZMUC), Muséum national d’Histoire naturelle, Paris, France (MNHN), and Institute of Biology and Soil Science, Far Eastern Branch, Russian Academy of Sciences, Vladivostok, Russia (IBSS), as indicated hereafter. Specimens were studied and illustrated using standard stereomicroscopic, photographic and drawing equipment.

In the catalogue sections, which mostly refer to the fauna of Taiwan alone, D stands for the original description, N for additional descriptive notes, and R for a mere mention or record.

## Systematics

### Nipponesmus shirinensis

Chamberlin & Wang, 1953

http://species-id.net/wiki/Nipponesmus_shirinensis

Figs 1 [Fig F2] 13

Nipponesmus shirinensis  Chamberlin & Wang, 1953: 4 (D).
Nipponesmus shirinensis  – Golovatch 1991: 157 (N); Korsós 2004: 26 (R)

#### Material examined:

2 juv. (NSYSU), Taiwan, Taipei City, Wenshan Distr., Chih-Nan Temple, 03.2002, leg. C.C. Chen et al.; 2 ♂, 2 ♀ (NMNS-6555-001), 1 ♂ (MNHN JC 331), Nantou County, Re-nai Township, Mei-Feng, 22.10.2001; 1 ♂ (ZMUC), 1 ♂ (NMNS-6555-002), same locality, 15.10.2001; 1 ♂ (TFRI), same locality, 19.02.2002; 1 ♂ (NMNS-6555-003), same locality, 15.10.2001; 1 ♂ (ZMUM), Nantou County, Huisun timber land, 20.09.1997; 1 ♂ (NMNS-6555-004), same locality, 10.1997; all leg. S.H. Wu; 3 ♂, 5 ♀ (TFRI), Nantou County, Lugu Township, Sitou, 15.11.2005, leg. J.D. Lee; 8 juv. (NSYSU), same locality, 6.07.2010, leg. H.W. Chang; 1 ♂, 1 ♀ (TFRI), Taitung County, Taimali Township, I-Ma forest road, 06.12.2004, leg. S.Y. Wu; 4 ♂, 1 ♀, 5 juv. (NSYSU), Chiayi County, Alishan Township, Nansi Forest road, ca 2,000 m a.s.l., 29.10.2010, leg. H.W. Chang; 1 ♂ (ZMUM), Hsinchu County, Wufeng Township, Sakaru (Shilu) trail, 30.09.2005; 1 ♂ (NMNS-6555-005), same locality, 22.09.2005, all leg. H.D. Zhu; 1 ♂ (NSYSU), Kaohsiung County, Liouguei, Shanping Workstation, 04.2004, leg. M.J. Hung; 1 ♂ (NMNS-6555-006), Kaohsiung County, Taoyuan Township, Tengjhih, 1,550 m a.s.l., 17.11.2010, leg. S. Golovatch; 2 ♀ (NMNS-6555-007), Kaohsiung County, Taoyuan Township, Chungchihkuan, 15.03.2005, leg. Y.C. Chang; 1 juv. (NSYSU), same locality, *Taiwania cryptomeroides* plantation, 15.10.2005, leg. M.H. Hsu; 1 ♂, 1 ♀ (IBSS), Pingtung County, Chunri Township, Mt Dahan, 1,200 m a.s.l., 15.12.2009; 2 ♀ (NMNS-6555-008), same locality, ca 1,850 m a.s.l., 15.12.2009, all leg. M.H. Hsu.

#### Diagnosis:

Differs from the other Polydesmidae in Taiwan by the larger body and often a dark coloration, from the only sympatric congener *Nipponesmus minor* sp. n. also in the collum being broader than the head and in the gonopod showing a strongly elongate, slender and falcate telopodite (see also Key below).

#### Redescription:

**Figures 1–5.**
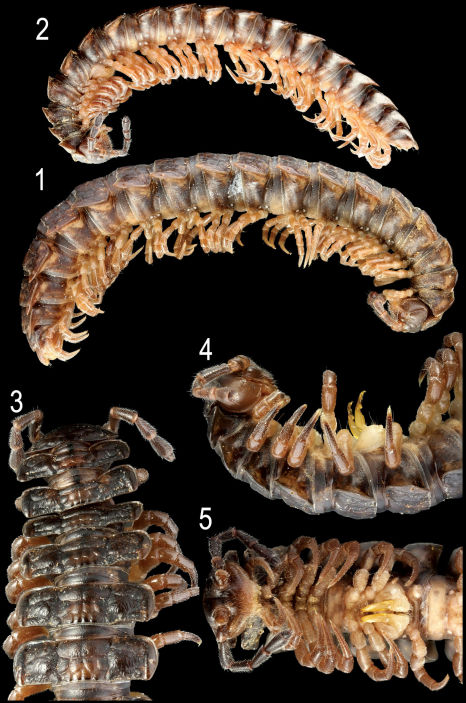
*Nipponesmus shirinensis* Chamberlin & Wang, 1953, ♀ (**1**) and ♂ (**2**) from Mei-Feng, ♂ (**3–5**) from Tengjhih. **1, 2** habitus, lateral view **3–5** anterior portion of body, dorsal, lateral and ventral views, respectively. Photographed not to scale.

**Figures 6–9. F2:**
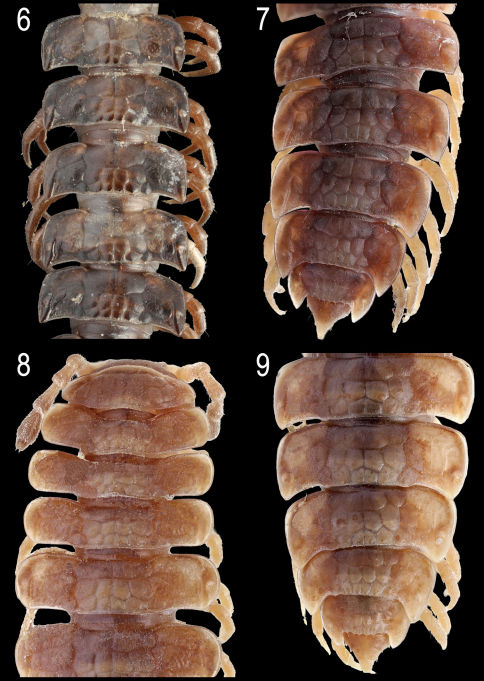
*Nipponesmus shirinensis* Chamberlin & Wang, 1953, ♂ (**6, 7**) from Tengjhih and ♀ (**8, 9**) from Mei-Feng. **6** middle portion of body, dorsal view **7, 9** posterior portion of body, dorsal view **8** anterior portion of body, dorsal view. Photographed not to scale.

Length of both sexes ca 18–25 mm, usually about 20–23 mm; width of pro- and metazona varying between specimens from 1.5–2.2 to 2.9–4.8 mm, respectively, usually 1.8–2.0 and 3.0–4.3 mm, respectively. Usually ♂♂ somewhat smaller than ♀♀. Coloration in alcohol from uniformly pallid (faded?) to dark chocolate-brown; in the latter case, sides, venter and legs (light) grey-brown to dark brown (Figs 1–6). Body with 20 segments. Tegument mainly dull, at most slightly shining, texture very delicately alveolate. Frons and labrum densely pilose, vertex bare; epicranial suture distinct but thin; a paramedian pair of evident, oblique ridges above antennal sockets; isthmus between antennae considerably broader than diameter of antennal socket (Fig. 5). Antennae rather long and only slightly clavate (Fig. 5), either slightly overreaching segment 3 dorsally (♂) or slightly shorter (♀); antennomere 3 longest (Figs 3–5); antennomeres 5 and 6 each with a small, compact, distodorsal group of bacilliform sensilla; antennomere 7 with a minute dorsoparabasal cone and a distodorsal group of microscopic sensilla.

In width, head < collum < segment 2 < 3 < 4 < 5=15(16), thereafter body gradually tapering towards telson. Paraterga strongly developed, starting from collum, subhorizontal, set high but always lying slightly below a faintly convex dorsum, drawn clearly forward only on metatergum 2 (Fig 3). Starting from segment 8 or 9 (♂) or 16 (♀), paraterga extending increasingly beyond rear tergal contour, caudal corners invariably evidently rounded, only on paraterga 17–19 subspiniform, usually slightly more narrowly rounded in ♂ compared to ♀ (Figs 3, 6–9). Paraterga more (♀) or less (♂) evidently rounded laterally (Figs 3, 6–9); starting from segment 2, all poreless segments with three, all pore-bearing ones with four, minute, sometimes even obliterate incisions at lateral margin. Front edges of metaterga slightly bordered and upturned, straight, usually forming a distinct shoulder. Pore formula normal, ozopores evident, dorsal, located in front of posteriormost marginal indentation. Metatergal sculpture typical, well-developed, with three transverse rows of setiferous, polygonal bosses (Figs 3, 6–9). Tergal setae very short, mostly obliterate, partly retained only on collum and/or metatergum 19. Stricture between pro- and metazona wide, shallow and smooth (Figs 3, 4, 6–9). Limbus very thin, microdenticulate. Pleurosternal carinae absent (Fig. 4). Epiproct rather short, conical, preapical papillae very evident (Figs 7, 9). Hypoproct semi-circular; caudal, paramedian, setiferous papillae small and well-separated.

**Figures 10–13.**
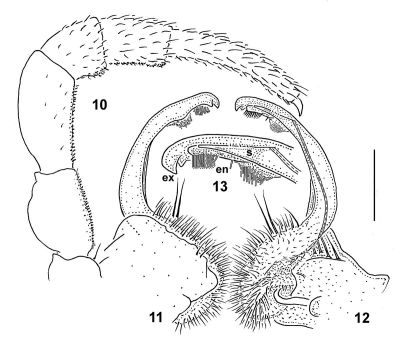
*Nipponesmus shirinensis* Chamberlin & Wang, 1953, ♂ from Sitou. **10** leg 9 **11, 12** right gonopod, lateral and mesal views, respectively **13** distal part of gonopod telopodite, enlarged, mesal view. Scale bar: 0.5 mm (**10–12**) and 0.25 mm (**13**). See text for explanation of labels.

Sterna without modifications, very densely (♂) (Fig. 5) or poorly (♀) setose, ♀ ones often shining. Epigynal ridge very low. Legs rather long and slender (Figs 5, 10), ca 1.5–1.6 (♂) or 1.1–1.2 (♀) times as long as midbody height; ♂ legs evidently enlarged, prefemora only slightly swollen dorsally and, like femora and, partly, postfemora, ventrally beset with shorter bifid setae turning into short sphaerotrichomes on postfemora, tibiae and tarsi (Fig. 10).

Gonopods (Figs 4, 5, 11–13) with large, subquadrate, medially fused coxae carrying a few long setae ventrally. Telopodite elongated, slender, falcate, prefemoral (densely setose) portion almost half as long as entire telopodite; seminal groove running mesally over most of its extent, only distally moving frontally to recurve first laterad and then a little basad at base of both a somewhat shorter, more complex endomere (**en**) and a longer, simpler exomere (**ex**); **en** beset with long, bacilliform setae distally and supplied with an evident spine (**s**) mesally, as well as a prominent pulvillus near base, this pulvillus being likewise beset with bacilliform setae and marking the end of seminal groove devoid of any accessory seminal chamber; **ex** strongly unciform apically, with an additional lateral tooth distally to subapically.

**Map 1.**
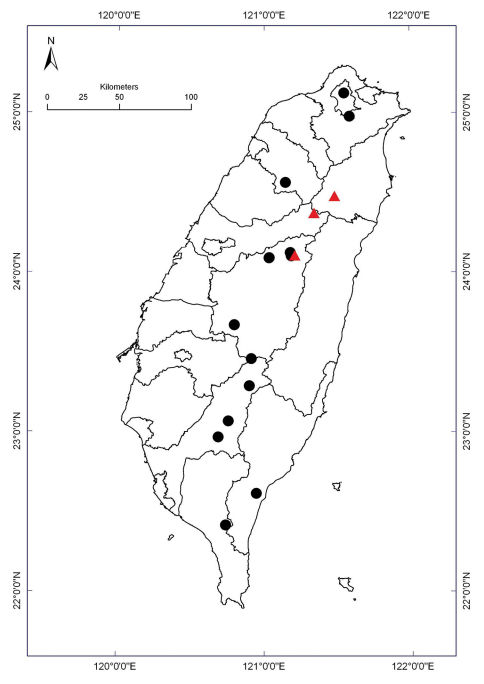
Distribution of *Nipponesmus* species in Taiwan. Borderlines show borders between the counties. *Nipponesmus minor* sp. n.: filled red triangles; *Nipponesmus shirinensis*: filled black circles.

**Remarks.** This species is the largest, as well as one of the most conspicuous and common among the polydesmids in Taiwan. It inhabits a wide range of habitats, mainly montane woodlands above 1,200 m a.s.l. (Map 1).

### Nipponesmus minor sp. n.

urn:lsid:zoobank.org:act:225E19E9-CF6B-46A1-A16F-7EFAD3A91A58

http://species-id.net/wiki/Nipponesmus_minor

Figs 14 23 

#### Type material:

Holotype ♂ (TFRI), Taiwan, Ilan County, Tatong Township, near Lakes Jialuohu, ca 2,250 m, 27.04.2003, leg. Y.M. Chen. Paratypes: 3 ♂, 1 ♀ (TFRI), same locality, 14.03.2003; 12 ♂, 8 ♀, 1 fragm. (TFRI), same locality, 28.04.2003; 1 ♂ (TFRI), same locality, 27.04.2003; 2 ♂ (ZMUM), 1 ♂ (IBSS), 2 ♂ (NSYSU), same locality, 25.12.2002; 1 ♂ (ZMUC), 1 ♂, 1 ♀ (TFRI), same locality, 7.11.2001; 1 ♂, 1 ♂ fragm. (TFRI), same locality, 4.06.2003, all leg. Y.M. Chen; 1 ♂ (ZMUM), same locality, 24.10.2002, leg. J.T. Chao; 1 ♂ (TFRI), Taichung County, Shengguang, 24.09.2002; 1 ♂ (TFRI), same locality, 24.01.2003; 1 ♀ (TFRI), same locality, 26.03.2003; 1 ♂ (MNHN JC 332), same locality, 24.01.2003; 1 ♂ (TFRI), same locality, 24.09.2002, all leg. W.C. Yeh; 1 ♂ (NMNS-6556-001), Nantou County, Ren-ai Township, Mei-Feng, 19.02.2002; 1 ♂ (NMNS-6556-002), same locality, 11.07.2002; 4 ♂ (NMNS-6556-003), same locality, 15.04.2002, all leg. S.H. Wu.

#### Name:

To emphasize the smaller body size and the shorter gonopod telopodite.

#### Diagnosis:

Differs from *Nipponesmus shirinensis*, the only other congener known from Taiwan, in the smaller size, as well as in the collum being narrower than the head and in the gonopod telopodite being stouter and shorter (see also Key below).

#### Description:

**Figures 14–19.**
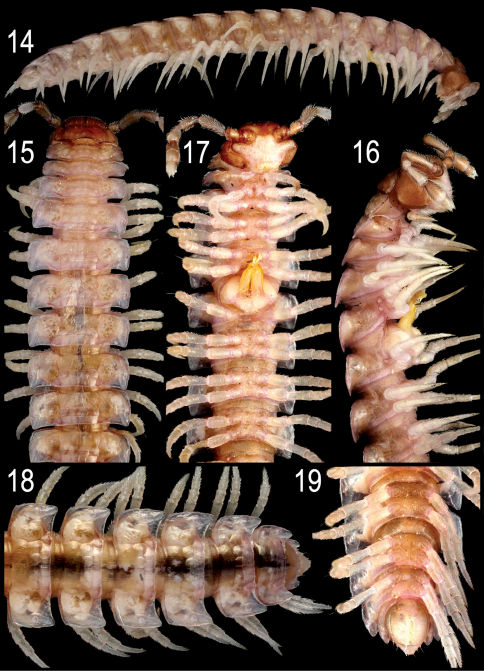
*Nipponesmus minor* **sp. n.**, ♂ paratype from Jialuohu. **14** habitus, lateral view **15–17** anterior portion of body, dorsal, lateral and ventral views, respectively **18, 19** posterior portion of body, dorsal and ventral views, respectively. Photographed not to scale.

Length of both sexes ca 12–16 mm; width of pro- and metazona varying between specimens from 0.8–1.3 to 1.5–2.0 mm, respectively. Holotype ca 12 mm long, and 0.8 and 1.5 mm wide on pro- and metazona, respectively. Usually ♂♂ somewhat smaller than ♀♀. Coloration in alcohol from uniformly pallid (faded?) to yellowish to reddish-brown; in the latter case, sides, venter and legs light grey-brown (Figs 14–19).

All characters as in *Nipponesmus shirinensis*, except as follows.

Antennae a little shorter, usually reaching midway of segment 3. In width, collum < head < segment 2 < 3 < 4 < 5=15(16), thereafter body gradually tapering towards telson. Starting from segment 16 (♂, ♀), paraterga extending increasingly beyond rear tergal contour, caudal corners invariably evidently rounded, but even on paraterga 17–19 not spiniform (Figs 15, 18). Paraterga less evidently rounded laterally even in ♀, lateral edges mostly subparallel in ♂ (Figs 15, 18), incisions being visible. Metatergal sculpture typical, rather superficial, with three transverse rows of setiferous, polygonal bosses (Figs 15, 18). Tergal setae very short, usually retained at least on collum and/or metatergum 19 (Figs 15, 16, 18).

**Figures 20–23.**
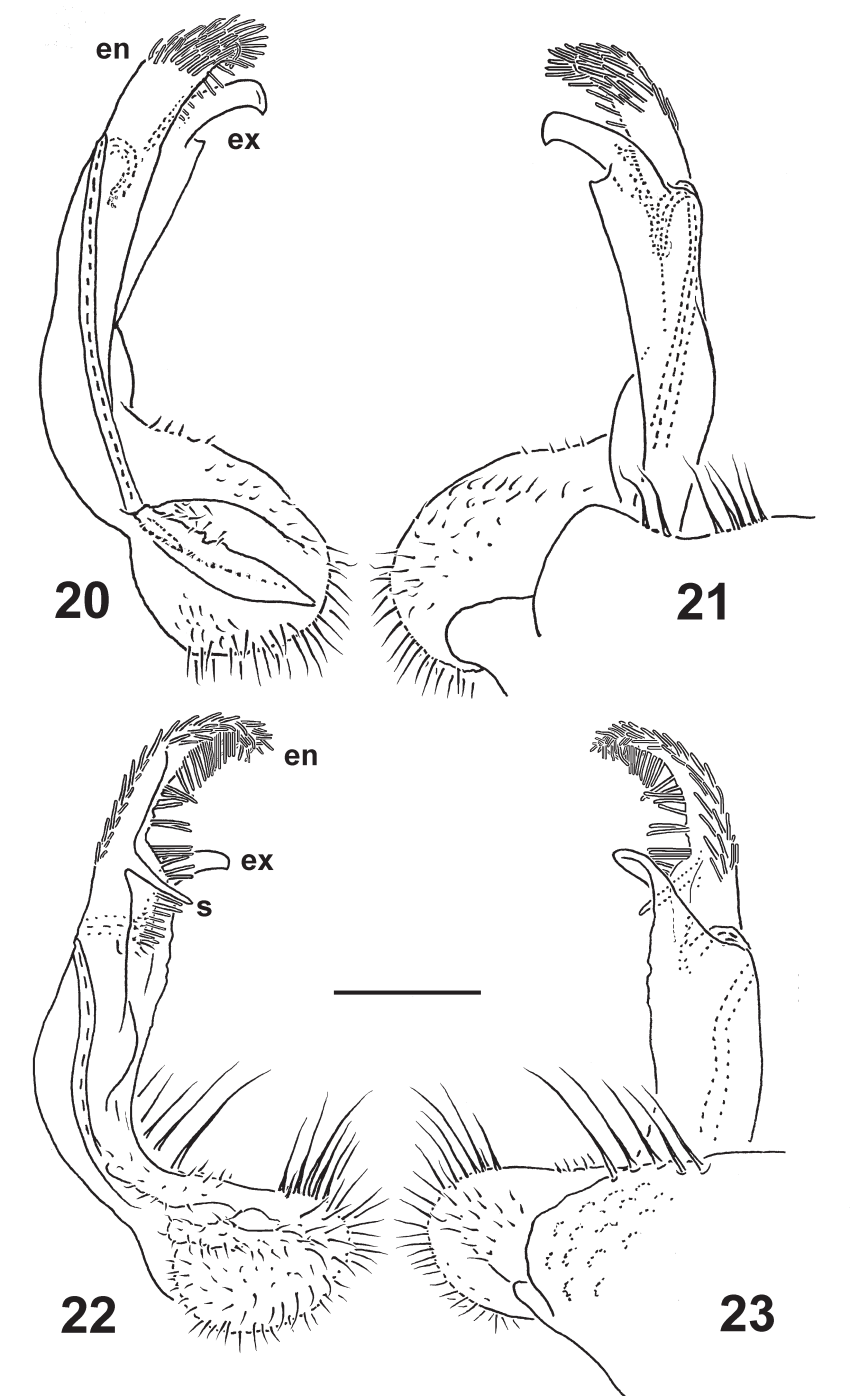
Left gonopods of *Nipponesmus minor* sp. n., ♂ paratypes from Mei-Feng (**20, 21**) and Jialuohu (**22, 23**), mesal, lateral, mesal and lateral views, respectively. Scale bar: 0.2 mm.

Gonopod telopodite (Figs 20–23) much stouter; endomere (**en**) slightly longer than exomere (**ex**), beset with long, bacilliform setae nearly throughout, usually supplied with an evident spine (**s**) or tooth mesally, as well as a usually somewhat less prominent pulvillus near base; **ex** often not so strongly unciform apically, often with an additional lateral tooth in distal part.

#### Remarks.

This species is not so widely distributed in Taiwan. Allopatry is prevailing, as it is only at Mei-Feng that both the congeners co-occur (Map 1). However, *Nipponesmus minor* sp. n. quite often lives even syntopically together with *Epanerchodus orientalis* (see below).

A study of the ample material representing not only the type species *Nipponesmus shirinensis*, but also the above new congener from Taiwan allows for the identity of *Nipponesmus* Chamberlin & Wang, 1953 to finally become clarified.

This genus has hitherto remained enigmatic, originally described too poorly (Chamberlin and Wang 1953) to shed any significant light on its affinities. It was only based on the gonopod conformation of the beautifully described *Nipponesmus tangonis* (Murakami, 1973), a species from Honshu, Japan definitely most similar to *Nipponesmus shirinensis*, that Golovatch (1991) suggested the relationships of *Nipponesmus* as a genus possibly somewhat intermediate between *Schizoturanius* Verhoeff, 1931 and *Epanerchodus* Attems, 1901.

In *Nipponesmus shirinensis*, the gonopod telopodite (Figs 11–13) is indeed biramous only distally, being divided into subequally prominent endo- and exomere. Furthermore, the endomere (**en**) is beset with a characteristically bacilliform trichome, whereas the exomere (**ex**) is simple. The seminal groove runs mostly mesally to recurve neatly between **ex** and **en** and then to debauch somewhat basally into a prominent hairy pulvillus which is also beset with the same peculiar trichome, and is devoid of an accessory seminal chamber. The same general pattern is observed both in *Nipponesmus tangonis* and *Nipponesmus minor* (Figs 20–23). What we term here as endomere is the branch which Murakami (1973) erroneously referred to as solenomere in his *Nipponesmus tangonis*. Yet it can hardly be called such, because the seminal groove ends somewhat basally of it and thus fails to support it at all. This is where one of the basic distinctions between *Nipponesmus* and *Schizoturanius* seems to lie, because in *Schizoturanius* the recurvature point of the seminal groove between both distal branches **ex** and **en** is about level to the pulvillus (Golovatch 1979). So we can speak in this case about a true solenomere. In addition, in *Schizoturanius* an accessory seminal chamber, however small, is present. Another characteristic feature of *Nipponesmus* vis-à-vis not only *Schizoturanius*, but also all other genera of Polydesmidae is an abundant bacilliform trichome on the endomere. Although a similar trichome is known to occur in some European *Polydesmus* species as well, it is always located either on the exomere or on a solenomere. These three apomorphies distinguish *Nipponesmus* from the obviously most similar *Schizoturanius*, a genus encompassing several species in Central Asia, Siberia and the southern part of the Eastern European Plain (Golovatch 1979, 1991).

As regards Golovatch’s (1991) placement of *Nipponesmus* also near *Epanerchodus*, following Hoffman (1980) who had synonymized these genera, this idea is false, as they appear to show too many profound differences in gonopod structure. Thus, in *Epanerchodus* the endomere is mostly absent, rarely present as only a rudimentary structure, while the seminal groove after the recurvature point still makes a long way basad to debauch into a prominent, simple-haired, accessory seminal chamber placed at the bottom of a profound parabasal cavity in the telopodite (see also below). Based on the gonopod conformation of one of the new Taiwanese species (see below), *Usbekodesmus* Lohmander, 1932, differing from *Epanerchodu*s only in a somewhat better developed exomere, albeit also simple and more or less spiniform, is to be regarded as another junior synonym of *Epanerchodus*, syn. n. Arguments for synonymizing both these genera have long been put forth (Geoffroy and Golovatch 2004), but until now no formal synonymy has been advanced.

This results in the following new formal transfers: *Epanerchodus redikorzevi* (Lohmander, 1932), from Uzbekistan, Tajikistan and Afghanistan, *Epanerchodus swatensis* (Golovatch, 1991), from Swat Province, northern Pakistan, *Epanerchodus varius* (Geoffroy & Golovatch, 2004), from Hubei Province, southern China, as well as the Nepalese *Epanerchodus anachoretus* (Golovatch, 1986), *Epanerchodus buddhis* (Golovatch, 1986), *Epanerchodus occultus* (Golovatch, 1986), *Epanerchodus sacer* (Golovatch, 1987), *Epanerchodus theocraticus* (Golovatch, 1990) and *Epanerchodus theosophicus* (Golovatch, 1986), all comb. n. ex *Usbekodesmus*.

The following key can serve to separate all three currently known species of *Nipponesmus*.

### Key to Nipponesmus species

**Table:** 

1	Adult body smaller, ≤ 10 mm long and ≤ 1.4 mm wide. Collum as wide as head. Endomere strongly enlarged, lobe-shaped, fringed by a very dense bacilliform trichome; exomere slender, much longer than endomere. Honshu, Japan	*Nipponesmus tangonis*
–	Adult body larger, at least 12 mm long and ≥ 1.5 mm wide. Collum either broader or narrower than head. Endomere at most slightly enlarged, digitiform, beset with a dense bacilliform trichome nearly all over; exomere rather short and stout, at most only slightly longer than endomere. Taiwan	
2	Adult body 18–25 mm long and 1.55–2.0 mm wide. Collum broader than head. Gonopod telopodite considerably longer (Figs 11–13)	*Nipponesmus shirinensis*
–	Adult body 12–16 mm long and 2.9–4.8 mm wide. Collum narrower than head. Gonopod telopodite considerably stouter (Figs 20–23)	*Nipponesmus minor* sp. n.

### Epanerchodus orientalis

Attems, 1901

http://species-id.net/wiki/Epanerchodus_orientalis

Figs 24 [Fig F8] 36

Epanerchodus orientalis orientalis  – Wang 1956: 155 (R); Wang 1963: 91 (R)
Epanerchodus orientalis takakuwai  – Wang 1958: 341 (R)

#### NB:

Complete catalogue information referring to this species can be found in Murakami (1969)

#### Material examined:

10 ♂ (TFRI), 1 ♂ (NMNS-6557-001), 1 ♀ (NSYSU), 2 ♂ (MNHN JC 333), Taiwan, Taichung County, Shengguang, 25.04.2003; 2 ♀ (TFRI), same locality, 26.03.2003; 1 ♀ (TFRI), same locality, 24.09.2002; 4 ♂ (TFRI), 1 ♂ (NMNS-6557-002), same locality, 24.07.2003, all leg. W.C. Yeh; 2 ♂, 1 ♀ (TFRI), 2 ♂, 1 ♀ (IBSS), Ilan County, Fushan, 23.03.2003; 2 ♀ (NMNS-6557-003), same locality, 18.05.2001; 5 ♂, 1 ♀ (TFRI), same locality, 19.06.2001; 1 ♂ (TFRI), same locality, without date, all leg. J.T. Chao; 1 ♂ (ZMUM), same locality, 20.11.2001, leg. S.S. Lu; 52 ♂, 17 ♀, 4 fragm. (TFRI), 2 ♂ (ZMUC), 2 ♂ (NSYSU), Ilan County, Tatong Township, near Lakes Jialuohu, ca 2,250 m, 4.06.2003; 1 ♀ fragm. (TFRI), same locality, 28.04.2003; 8 ♂, 6 ♀ (TFRI), same locality, 20.06.2002; 1 ♂ (ZMUM), same locality, 25.04.2003; 1 ♂ (TFRI), same locality, 23.07.2002, all leg. Y.M. Chen; 1 ♀ (NSYSU), Taitung County, Yanping Township, Yanping Forest road, ca. 1,250 m, 3.06.2003, leg. M.H. Hsu; 2 ♂ (NSYSU), Hualien County, Fongbin Township, Guangfong Highway, ca 300 m, 6.05.2009; 1 ♀ (NSYSU), same County, Shoufang Township, ca. 230 m a.s.l., 5.05.2009, all leg. M.H. Shu; 2 ♀ (NMNS-6557-004), Pingtung County, Chaozhou Township, Si-Lin, 1.06.1999, leg. ?; 1 ♀ (NMNS-6557-005), Kaohsiung County, Taoyuan, Provincial Road No. 20, near 142.5 km road-sign, 3.09.2009, leg. C.Y. Huang.

#### Diagnosis:

Differs from the other *Epanerchodus* species known from Taiwan in the variable, mostly medium size, coupled with the caudal corner of most of the paraterga being pointed, and in the gonopod telopodite being relatively slender, complex, highly variable in shape and armature (see also Key below).

#### Redescription:

**Figures 24–27.**
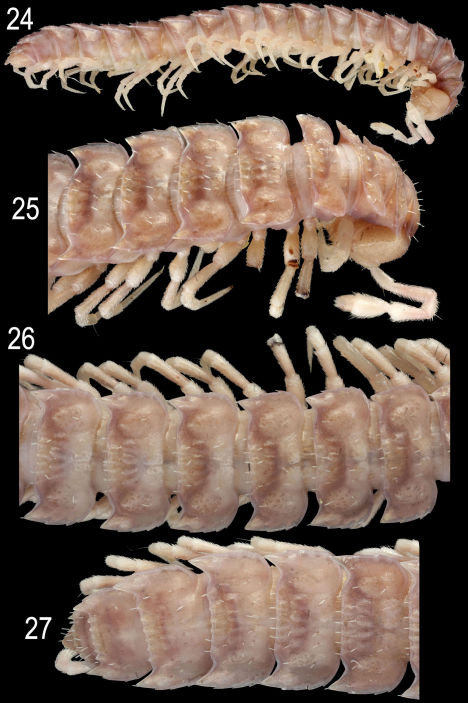
*Epanerchodus orientalis* Attems, 1901, ♂ from Fushan. **24** habitus, lateral view **25** anterior portion of body, subdorsal view **26** middle portion of body, dorsal view **27** posterior portion of body, dorsal view. Photographed not to scale.

**Figures 28–31. F8:**
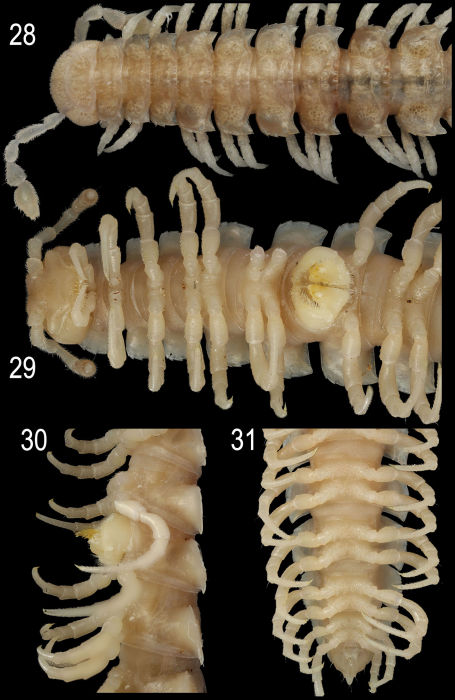
*Epanerchodus orientalis* Attems, 1901, ♂ from Jialuohu. **28, 29** anterior portion of body, dorsal and ventral views, respectively **30** middle portion of body, lateral view **31** posterior portion of body, ventral view. Photographed not to scale.

Length of both sexes ca 11–19 mm; width of pro- and metazona varying between specimens from 0.8–1.8 to 1.2–3.0 mm, respectively. Usually ♂♂ somewhat smaller than ♀♀. Coloration in alcohol from uniformly pallid (faded?) to light (reddish- to grey-) brown; in the latter case, venter and legs light grey to yellow (Figs 24–31). Body with 20 segments. Tegument mainly dull, at most slightly shining, texture very delicately alveolate. Labrum, frons and vertex densely pilose; epicranial suture clear but thin; no ridges above antennal sockets; isthmus between antennae considerably broader than diameter of antennal socket (Fig. 29). Antennae long and only slightly clavate (Figs 24, 25, 28), reaching behind end (♂) or midway (♀) of segment 4 dorsally; antennomere 3 longest, 5th highest (Figs 25, 28); antennomeres 5 and 6 each with a small, compact, distodorsal group of bacilliform sensilla; antennomere 7 with a minute dorsoparabasal cone and a distodorsal group of microscopic sensilla.

In width, either collum ≤ segment 2 = 3 < 4 < head = 5(6)-15(16), or segment 2 = 3 < collum < head = 4 < 5–15(16) (both sexes), thereafter body gradually tapering towards telson. Paraterga strongly developed, starting from collum, subhorizontal, set high but always lying slightly below a faintly convex dorsum; front shoulders and caudal edge nearly straight on several postcollum metaterga, caudal corner always pointed. Collum (Fig. 28) nearly regularly elliptical, with an incision near caudal corner and three transverse rows of setae (6+6, 4+4 and 3+3). Starting from segment 5, paraterga extending increasingly beyond rear tergal contour, mostly subspiniform (Figs 25–28). Starting from segment 2, all poreless segments with three, all pore-bearing ones with four, small but evident incisions, each usually bearing a small seta on top at lateral margin. Pore formula normal, ozopores evident, dorsal, located in front of 4th indentation. Metatergal sculpture typical, rather well developed, with three transverse rows of setiferous, polygonal bosses (Figs 25–28). Tergal setae short, mostly retained, a little longer only on collum and in rear row on metatergum 19 (Figs 25, 27). Stricture between pro- and metazona rather obscure, wide, smooth and polished. Limbus very thin, microdenticulate. Pleurosternal carinae absent. Epiproct rather short, conical (Fig. 31), preapical papillae evident. Hypoproct semi-circular; caudal, paramedian, setiferous papillae evident and well-separated.

Sterna without modifications (Figs 29, 31), densely setose. Epigynal ridge very low, regularly rounded. Legs long and slender (Figs 28, 29), ca 1.8–1.9 (♂) or 1.2–1.3 (♀) times as long as midbody height; ♂ legs evidently enlarged, prefemora only slightly swollen dorsally, femora ventro-parabasally with shorter bifid setae replaced by sphaerotrichomes in remaining parts of femora, as well as on entire postfemora, tibiae and tarsi.

**Figures 32–36.**
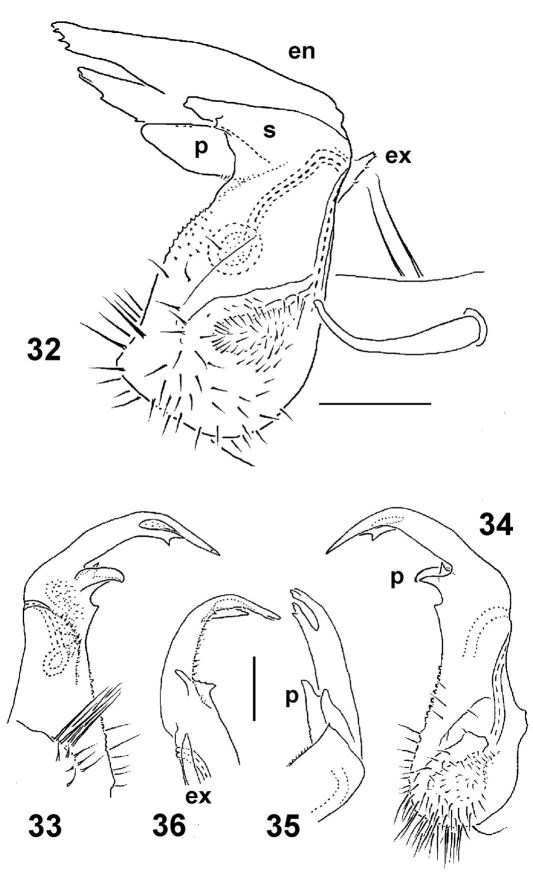
Right gonopods of *Epanerchodus orientalis* Attems, 1901, ♂♂ from Shengguang (**32**), Jialuohu (**33–35**) and Guangfong Highway (**36**), mesal, lateral, mesal, sublateral and submesal views, respectively. Scale bar: 0.1 mm.

Gonopods (Figs 32–36) with large, subquadrate, medially fused coxae carrying a few long setae ventrally. Telopodite stout to rather slender, subfalcate, prefemoral (densely setose) portion one-third to half as long as entire telopodite; seminal groove running mesally over much of its extent, only distally moving frontally to recurve first laterad and then mesad, squeezing neatly between a simple, more or less rudimentary to completely reduced exomere (**ex**) and a more complex, branching and always well-developed endomere (**en**), then groove continuing to a considerable extent basad to end into a large accessory seminal chamber lying near base of a prominent excavation, the latter carrying an evident hairy pulvillus; **en** often but not alwayswith a strong mesal process (**s**), either slenderer and simple or larger and more complex in shape, as well as usually with a distinct, often again branching, laterobasal outgrowth (**p**); distal remainder of **en** elongate, often branching and enlarged, sometimes fringed with short setae or spines.

#### Remarks.

This species seems to be the most widespread and variable among congeners. It has heretofore been accepted as being split into two nominal subspecies: *Epanerchodus orientalis orientalis* and *Epanerchodus orientalis takakuwai* Verhoeff, 1913, but, given a similarly profound variation range in body size and shape, and, especially, in gonopod structure as observed in Japan alone, the subspecific status of *takakuwai* has long been questioned (e.g. Miyosi 1959, Murakami 1969, Nishikawa and Murakami 1993). Murakami (1993), in the latest checklist of the Japanese Diplopoda, treats *Epanerchodus orientalis* without subspecies. Moreover, numerous samples from Hokkaido, Honshu and Shikoku, Japan, reveal that variation in gonopod conformation appears to be purely individual, failing to demonstrate any meaningful geographical patterns (Nishikawa and Murakami 1993). The same concerns the available samples from Taiwan. So we formalize here the long suspected synonymy: *Epanerchodus orientalis orientalis* Attems, 1901 = *Epanerchodus orientalis takakuwai* Verhoeff, 1913, syn. n.

It is noteworthy that, formally, *Epanerchodus orientalis* fails to occur in southernmost Japan, i.e. on Kyushu Island and in the Ryukyus (Nishikawa and Murakami 1993, Nakamura and Korsós 2010), to reappear further south only in Taiwan. In northern Honshu, Japan, it is known to reach 26 mm in length and 3.4 mm in width, with the collum being slightly broader than the head (Murakami 1969). In Taiwan, as proven by our study of several abundant syntopic samples, the animals tend to be smaller, the collum is invariably narrower than the head, while rather often the gonopods are totally devoid of both an exomere remnant and process **s**, frequently with the distal, longest part of their endomere being slender, not expanded.

Looking at such a profound variation as observed in a number of peripheral and gonopod characters in *Epanerchodus orientalis*, one cannot ignore several further nominal congeners described from Japan, including Kyushu, in which the gonopods look especially similar to those of *Epanerchodus orientalis*: *Epanerchodus inferus* Verhoeff, 1941, *Epanerchodus lobatus* Verhoeff, 1941, *Epanerchodus satoi* Takakuwa, 1954, *Epanerchodus tenuis* Takakuwa, 1954, *Epanerchodus aculeatus* Miyosi, 1954, *Epanerchodus etoi* Miyosi, 1955, *Epanerchodus chichibensis* Haga, in Takashima and Haga 1956, *Epanerchodus yoshidai* Haga, in Takashima and Haga 1956, *Epanerchodus lacteus* Shinohara, 1958, etc. (e.g. Takashima and Haga 1956, Shinohara 1958, Miyosi 1959). We suspect that some of them might well prove to represent junior synonyms of *Epanerchodus orientalis*. Only future in-depth biological observations and such genetic investigations as bar-coding of such particularly similar forms can shed light on their true identities and statuses, because polymorphic variation has long been known in *Epanerchodus*. Thus, *Epanerchodus polymorphus* Mikhaljova & Golovatch, 1981, widespread in the southern part of the Russian Far East and in northern Korea, shows two morphologically distinct morphs both in gonopod and peripheral structure in males, but a complete, overlapping range of the same somatic characters in females. Both male morphs invariably co-occur syntopically and either mates with any female variety (Mikhaljova and Golovatch 1981, Mikhaljova 2004). A similar situation is found in the nominate species *Epanerchodus acuticlivus* Murakami, 1970 and *Epanerchodus aster* Murakami, 1970, both described from the same cave in Shikoku, Japan. Murakami (1970) explicitly admitted that they were very close, with their females being indistinguishable, while the males showed small but stable differences in gonopod telopodite armature.

Based on the great variation observed in the populations of *Epanerchodus orientalis* in Japan and, especially, Taiwan, the statuses of *Epanerchodus polymorphus*, *Epanerchodus acuticlivus* and *Epanerchodus aster* as further congeners highly similar to *Epanerchodus orientalis* are likewise to be questioned, as at least some of them might also prove to be the latter’s junior synonyms. Yet no formal synonymies are advanced here as obviously being too premature at this purely descriptive taxonomic stage.

That not all of the congeners similar in gonopod structure to *Epanerchodus orientalis* are synonyms of the latter is proven at least by *Epanerchodus koreanus* Verhoeff, 1937, a species common throughout Korea, in Kyushu, Japan and in the southern Russian Far East. At least in Russia, it often occurs syntopically together with *Epanerchodus polymorphus*. Yet *Epanerchodus koreanus*, despite its minor variations in gonopod conformation, is easily recognizable even superficially through its much wider paraterga (3.3–3.8 versus 2.6–3.0 mm); in addition, these species never mate (Mikhaljova 2004). As another proof to the above statement may also serve *Epanerchodus pinguis* sp. n., described below and representing still one more congener quite similar in gonopod structure to *Epanerchodus orientalis*. In Taiwan, both are at least partly sympatric, but *Epanerchodus pinguis* sp. n. differs markedly enough in a number of peripheral and gonopod characters to warrant recognition of a distinct species (see below).

In other words, several, but definitely not all, of the nominate species of *Epanerchodus* that show their gonopods particularly similar in structure to those of *Epanerchodus orientalis* are jeopardized as potential junior synonyms of the latter species.

**Map 2.**
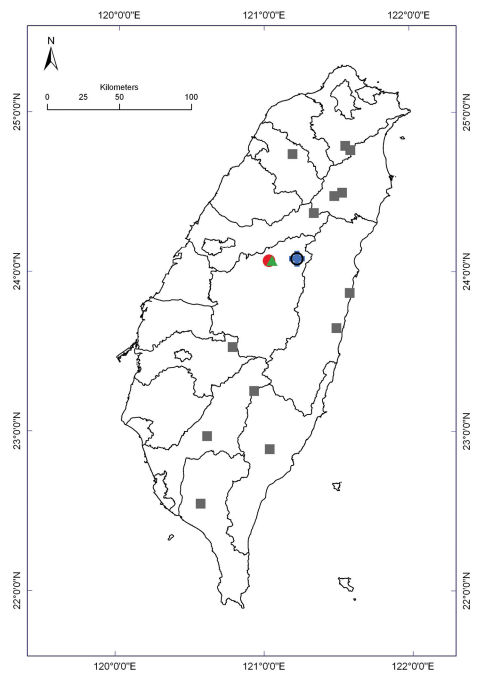
Distribution of *Epanerchodus* species in Taiwan. Borderlines show borders between the counties. *Epanerchodus bispinosus* sp. n.: empty black circle; *Epanerchodus curtigonopus* sp. n.: blue cross; *Epanerchodus flagellifer* sp. n.: filled green triangles; *Epanerchodus orientalis*: filled grey squares; *Epanerchodus pinguis* sp. n.: filled red circle.

In Taiwan, *Epanerchodus orientalis* occurs in various habitats ranging from lowlands to the mid-montane belt (up to 1,250 m a.s.l.) all over the island (Map 2).

### Epanerchodus pinguis sp. n.

urn:lsid:zoobank.org:act:B0E8A6F1-335A-4961-9876-0328480D6D47

http://species-id.net/wiki/Epanerchodus_pinguis

Figs 37 44 

#### Type material:

Holotype ♂ (head missing) (NMNS-6558-001), Taiwan, Nantou County, Huisun timber land, 15.04.2002, leg. S.H. Wu. Paratype ♂ (NMNS-6558-002), same locality, 24.03.1998, leg. S.H. Wu.

#### Name:

To emphasize the stout gonopod telopodite.

#### Diagnosis:

Differs from the other *Epanerchodus* species, in particular from the apparently especially similar *Epanerchodus orientalis*, in the mostly square, broader and slightly upturned paraterga, coupled with the gonopod showing an unusually densely setose coxa and a remarkably stout telopodite (see also Key below).

#### Description:

**Figures 37–41.**
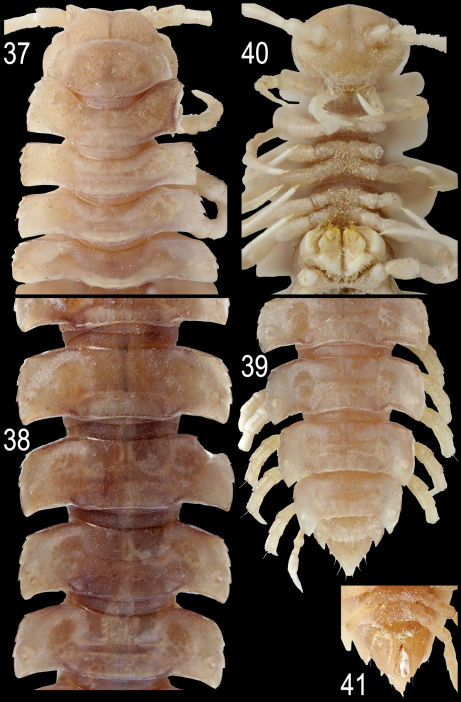
*Epanerchodus pinguis* sp. n., ♂ paratype. **37, 40** anterior portion of body, dorsal and ventral views, respectively; **38**, middle portion of body, dorsal view **39, 41** posterior portion of body, dorsal and ventral views, respectively. Photographed not to scale.

Length ca 16 (paratype) or 20 mm (holotype); width of midbody pro- and metazona 1.5 and 2.9 mm (paratype) or 1.5 and 3.0 mm (holotype), respectively. Coloration in alcohol uniformly light red-brown to red-brown; venter and legs yellowish (Figs 37–39).

All characters as in *Epanerchodus orientalis* except as follows.

Antennae rather long, slender, only slightly clavate, reaching behind segment 3 dorsally; antennomere 3 longest, clearly longer than highest 5th; antennomeres 5 and 6 each with an evident, compact, distodorsal group of bacilliform sensilla; antennomere 7 with a minute dorsoparabasal cone and a distodorsal group of microscopic sensilla.

In width, collum < head ≤ segment 2 ≤ 3 < 4 < = 5–16, thereafter body gradually tapering towards telson (Fig. 39). Paraterga strongly developed, starting from collum, slightly upturned, set high, mostly level to a very faintly convex dorsum, slightly below dorsum only on collum and segment 2; paraterga on collum small, subtriangular, a small lateral incision in front of a narrowly rounded caudal corner; front shoulders drawn forward only paraterga 2 and 3, straight and subrectangular on paraterga 4, onward straight but directed increasingly caudolaterad; caudal edge nearly straight on paraterga 2–7, thereafter caudal corner increasingly acutangular, nearly always narrowly rounded, only on segment 19 spiniform and nearly pointed, mostly lying nearly level to rear tergal contour until segment 15, onward extending increasingly beyond it (Figs 37–39). Paraterga 2 with 3–4 small lateral incisions, all following poreless segments with three, all pore-bearing ones with four, small but evident incisions, each usually bearing a small seta on top at lateral margin. Metatergal sculpture typical, rather obliterate, with three indistinct transverse rows of setiferous, polygonal bosses (Figs 37–39). Tergal setae very short, mostly retained, a little longer only on collum and in rear row on metatergum 19 (Figs 37, 39). Stricture between pro- and metazona wide and smooth. Limbus very thin, microdenticulate. Epiproct rather short, conical (Fig. 39), preapical papillae prominent. Hypoproct semi-circular; caudal, paramedian, setiferous papillae evident and well-separated.

**Figures 42–44.**
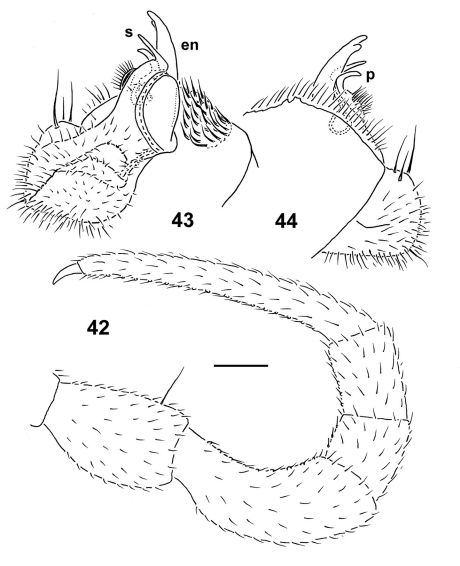
*Epanerchodus pinguis* sp. n., holotype. **42** leg 9 **43, 44** right gonopod, mesal and lateral views, respectively. Scale bar: 0.2 mm.

Sterna without modifications, very densely setose (Figs 40, 41). Legs long and slender, evidently enlarged (Figs 42), ca 1.6–1.7 times as long as midbody height, prefemora not swollen dorsally, acropodite (femur+postfemur+tibia+tarsus) with sphaerotrichomes ventrally (Fig. 42).

Gonopods (Figs 40, 43, 44) with large, subquadrate, medially fused coxae carrying numerous long setae ventromedially. Telopodite mostly hidden inside gonocoel, unusually stout, subfalcate, prefemoral (densely setose) portion about half as long as entire telopodite; endomere (**en**) short, simple, with a subapical knob laterally and a rounded tip, as well as with two subunciform processes (**s** and **p**); hairy pulvillus very evident, exomere totally suppressed.

#### Remarks.

This species is apparently very local in distribution (Map 2) and seems to be allopatric with *Epanerchodus orientalis*.

### Epanerchodus bispinosus sp. n.

urn:lsid:zoobank.org:act:5944975E-495B-4578-8145-779D40B9C80A

http://species-id.net/wiki/Epanerchodus_bispinosus

Figs 45 53 

#### Type material:

Holotype ♂ (NMNS-6559-001), Taiwan, Nantou County, Ren-ai Township, Mei-Feng, 15.04.2002, leg. S.H. Wu. Paratypes: 3 ♂, 1 ♂ fragm., 4 ♀, 1 ♀ juv. (NMNS-6559-002), 1 ♂, 1 ♀ (ZMUM), 1 ♂ (without head), 1 ♀ (ZMUC), 1 ♂ fragm., 1 ♀ (MNHN JC 334), 1 ♂ (TFRI), 1 ♂ (NSYSU), same locality, together with holotype.

#### Name:

To emphasize the distal half of the gonopod telopodite being like two long spines.

#### Diagnosis:

Due to the presence of a strong and spiniform exomere, this new species joins the few congeners hitherto referred to the erstwhile genus *Usbekodesmus* (see above the synonymy with *Epanerchodus*), but differs in the distal half of the gonopod telopodite being represented by only two spiniform branches showing no additional outgrowths (see also Key below).

#### Description:

**Figures 45–50.**
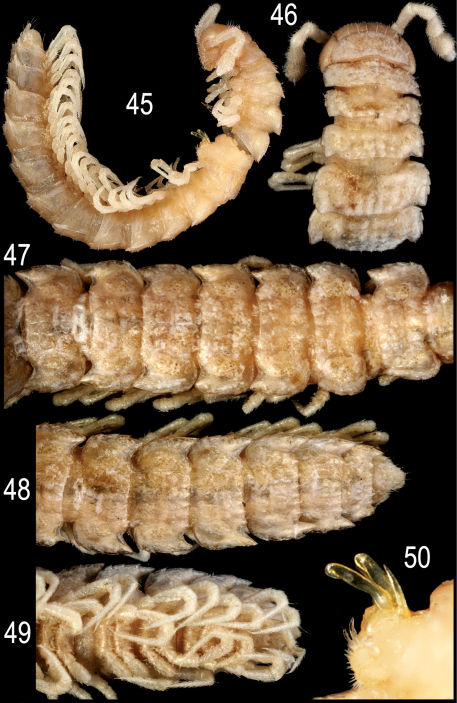
*Epanerchodus bispinosus* sp. n., ♂ paratype. **45** habitus, lateral view **46** anterior portion of body, dorsal view **47** middle portion of body, dorsal view **48, 49** posterior portion of body, dorsal and ventral views, respectively **50** gonopods, lateral view. Photographed not to scale.

Length of both sexes ca 8–11 mm; width of pro- and metazona varying between specimens from 0.8–1.3 to 1.1–1.7 mm, respectively. Holotype ca 9 mm long, and 0.8 and 1.1 mm wide on pro- and metazona, respectively. Coloration in alcohol from pallid to uniformly light grey, yellow or very light red-brown, sometimes head faintly marbled light red-brown; venter and legs yellowish to greyish (Figs 45–49).

All characters as in *Epanerchodus orientalis* except as follows.

Antennae rather long and evidently clavate, reaching behind end (♂) or midway (♀) of segment 3 dorsally; antennomere 3 longest, considerably longer than a relatively stout, yet highest, 5th (Figs 45); antennomeres 5 and 6 each with a small, compact, distodorsal group of bacilliform sensilla; antennomere 7 with a minute dorsoparabasal cone and a distodorsal group of microscopic sensilla.

In width, collum < segment 2 = 3 < 4 < head = 5–15 (Fig. 46), thereafter body gradually tapering towards telson (♂, ♀) (Fig. 48). Paraterga moderately developed, starting from collum, set high but invariably lying slightly below a faintly convex dorsum; paraterga on collum small, subtriangular, with a small lateral incision in front of a rounded caudal corner; front shoulders drawn forward and slightly convex only paraterga 2–4, thereafter straight, increasingly well rounded and directed increasingly caudolaterad; caudal corner on pataterga 2–5 subrectangular and narrowly rounded, starting from 6th increasingly acutangular and beak-shaped, starting from segment 9 first faintly and then extending increasingly beyond rear tergal contour (Figs 45–48). All poreless segments with three, all pore-bearing ones with four, small but evident incisions, each usually bearing a small seta on top at lateral margin. Metatergal sculpture typical, moderately developed, with three indistinct transverse rows of setiferous, polygonal bosses (Figs 46–48). Tergal setae very short, mostly retained, a little longer only on collum and in rear row on metatergum 19. Stricture between pro- and metazona wide and smooth. Limbus very thin, microdenticulate. Epiproct rather short, conical (Fig. 48), only slightly bent ventrad, preapical papillae evident. Hypoproct semi-circular; caudal, paramedian, setiferous papillae evident and well-separated.

**Figures 51–53.**
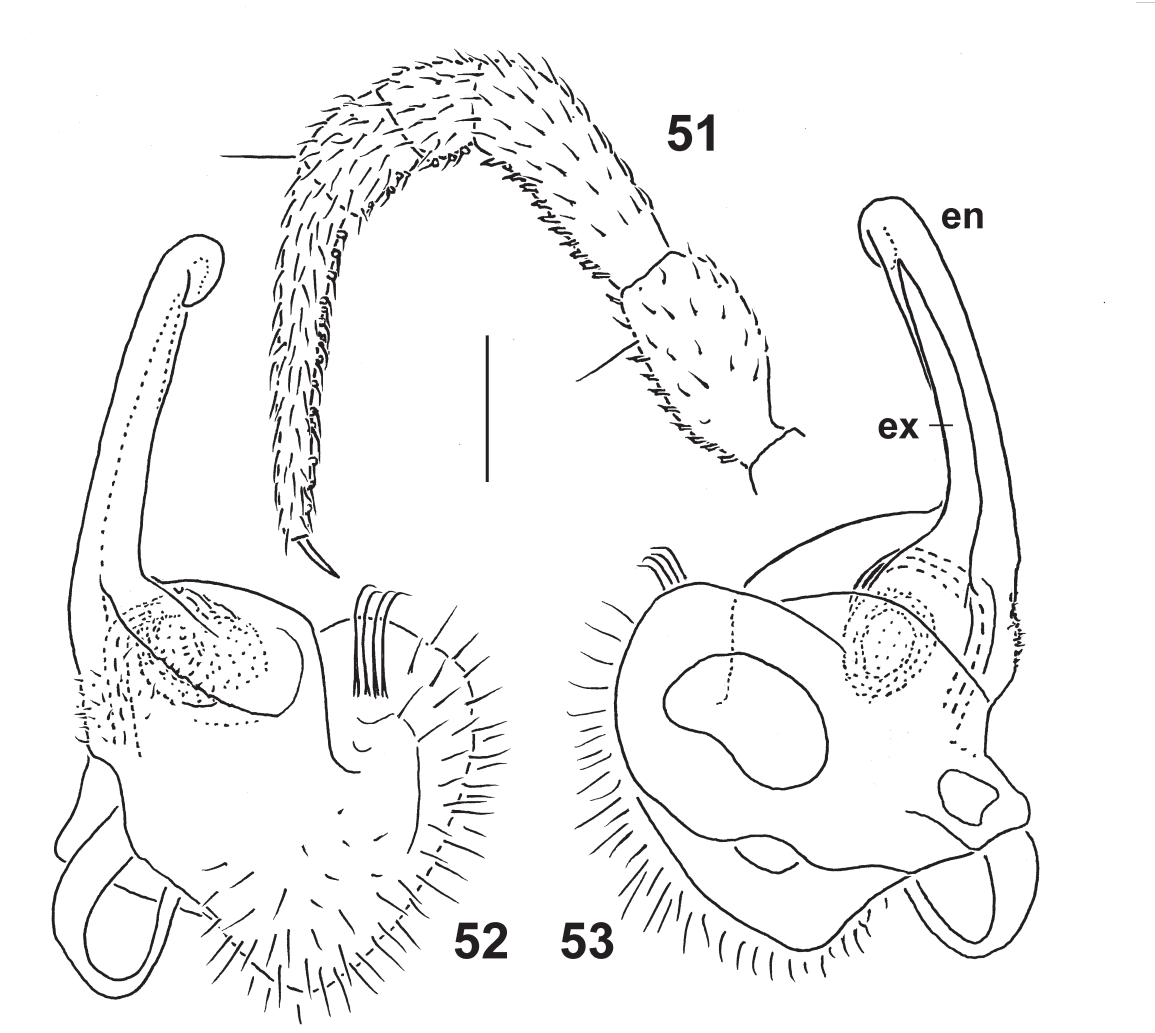
*Epanerchodus bispinosus* sp. n., ♂ paratype. **51** leg 9 **52, 53** left gonopod, submesal and sublateral views, respectively. Scale bar: 0.4 mm (**51**) and 0.2 mm (**52, 53**).

Sterna without modifications, very densely setose. Legs rather short, slender, in ♂ evidently enlarged (Figs 45), ca 1.5–1.6 (♂) or 1.1–1.2 times (♀) as long as midbody height, ♂ prefemora not swollen dorsally, together with femora beset with bifid setae ventrally, acropodite (postfemur+tibia+tarsus) with sphaerotrichomes ventrally (Fig. 51).

Gonopods (Figs 50, 52, 53) with large, subquadrate, medially fused coxae carrying only a few long setae ventrally. Telopodite slender and simple, prefemoral portion relatively short, exomere (**ex**) spiniform, endomere (**en**) only a little longer than **ex**, apex bent unciform mesally, devoid of any outgrowths near base; hairy pulvillus evident.

#### Remarks.

This species is remarkable in showing an exomere, albeit usual and spiniform, almost as long as a particularly simple endomere. This condition nicely bridges the weak distinction which has hitherto remained to formally keep *Usbekodesmus* and *Epanerchodus* as independent genera (Geoffroy and Golovatch 2004), with the gonopod structure in *Epanerchodus bispinosus* sp. n. providing the final evidence to formally synonymize these genera (see also above).

*Epanerchodus bispinosus* sp. n. in Taiwan is apparently very local in distribution, having been encountered only at a single locality (Map 2).

### Epanerchodus curtigonopus sp. n.

urn:lsid:zoobank.org:act:CBDF0100-0AEF-4D12-B88A-3A59119AD5C0

http://species-id.net/wiki/Epanerchodus_curtigonopus

Figs 54 60 

#### Type material:

Holotype ♂ (NMNS-6560-001), Taiwan, Nantou County, Ren-ai Township, Mei-Feng, 15.10.2001, leg. S.H. Wu.

#### Name:

To emphasize the remarkably short gonopod telopodite.

#### Diagnosis:

Differs from other *Epanerchodus* species in the distal half of the gonopod telopodite being particularly stout, with the endomere represented only by a short spine supplied with a similarly short spine at its base (see also Key below). This new species seems to be particularly similar to *Epanerchodus pinguis* sp. n. in sharing the gonopod which shows a densely setose coxa and a very stout telopodite (apparently, both being synapomorphies), but differs in the entire leg telopodites (prefemur+femur+postfemur+tibia+tarsus) being supplied with sphaerotrichomes ventrally, and the endomere and its basal process rudimentary and spiniform.

#### Description:

Length ca 15 mm; width of pro- and metazona 1.3 and 2.5 mm, respectively. Coloration in alcohol pallid.

Superficially, also very similar to *Epanerchodus pinguis* sp. n., except as follows.

**Figures 54–57.**
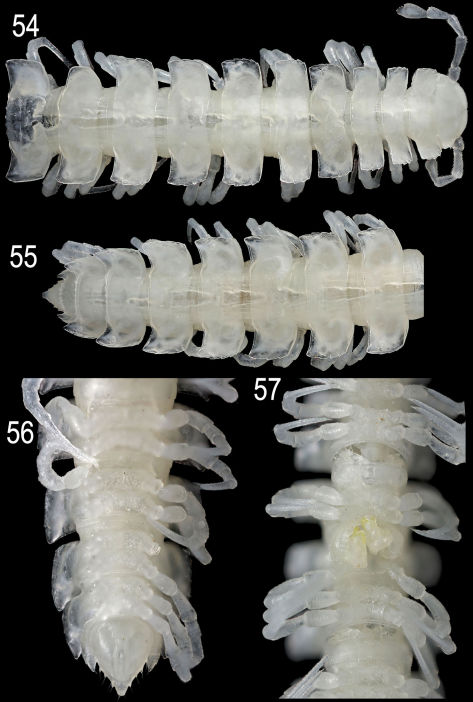
*Epanerchodus curtigonopus* sp. n., holotype. **54** anterior portion of body, dorsal view **55, 56** posterior portion of body, dorsal and ventral views, respectively **57** middle portion of body (with gonopods), ventral view. Photographed not to scale.

In width, collum < head < segment 2 = 3 < 4 < 5 < 6–15 (Figs 54), thereafter body gradually tapering towards telson (Fig. 55). Paraterga strongly developed, starting from collum, set high, subhorizontal to very faintly upturned, lying slightly below dorsum only on collum and segment 2; mid-dorsum invariably extremely faintly convex, nearly flat; front shoulders mostly straight, drawn forward only on paraterga 2 and 3, thereafter directed increasingly caudolaterad and increasingly well rounded anterolaterally; caudal corner on paraterga 3–12 subrectangular and narrowly rounded, starting from 13th increasingly acutangular and evidently extending increasingly beyond rear tergal contour, but nearly pointed and beak-shaped only on segment 19 (Figs 54, 55). Paraterga 2–5 and all following pore-bearing segments with four, following poreless ones with three, small but evident incisions, each usually bearing a small seta on top at lateral margin. Metatergal sculpture typical, moderately developed, with three indistinct transverse rows of setiferous, polygonal bosses (Figs 54, 55). Tergal setae very short, mostly retained, a little longer only on collum and in rear row on metatergum 19. Stricture between pro- and metazona wide and smooth. Limbus very thin, microdenticulate. Epiproct rather short, conical (Figs 55, 56), only slightly bent ventrad, preapical papillae evident. Hypoproct semi-circular; caudal, paramedian, setiferous papillae distinct and well-separated (Fig. 56).

**Figures 58–60.**
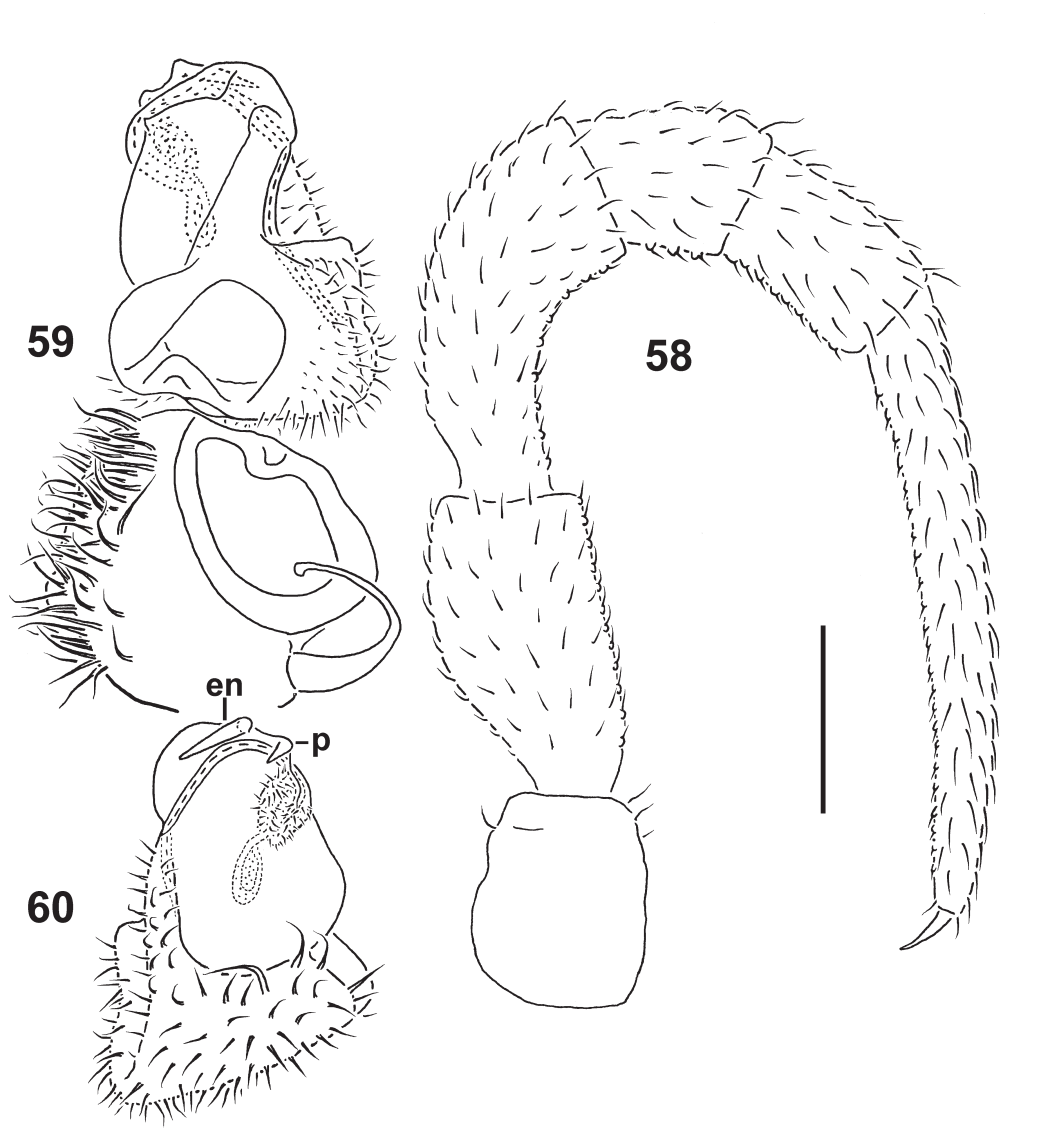
*Epanerchodus curtigonopus* sp. n., holotype. **58** leg 9 **59, 60** left gonopod, dorsomesal and ventrolateral views, respectively. Scale bar: 0.2 mm.

Sterna without modifications, densely setose. Legs long and rather slender, only slightly enlarged (Figs 56, 57), ca 1.7–1.8 times as long as midbody height, prefemora not swollen dorsally, entire telopodite (prefemur+femur+postfemur+tibia+tarsus) with sphaerotrichomes ventrally (Fig. 58).

Gonopods (Figs 57, 59, 60) with large, subquadrate, medially fused coxae carrying numerous setae ventrally. Telopodite especially stout, prefemoral portion relatively prominent, taking up about one-third of telopodite length, endomere (**en**) like a short spine supplied with another, somewhat shorter spine (**p**) near base; both accessory seminal chamber and hairy pulvillus evident.

#### Remarks.

This species is remarkable in showing an extremely short, simple, spiniform endomere (**en**), coupled with a single, even more rudimentary process (**p**) at **en** base.

In Taiwan, *Epanerchodus curtigonopus* sp. n. occurs very locally, having been found only at a single locality (Map 2).

### Epanerchodus flagellifer sp. n.

urn:lsid:zoobank.org:act:35EAB5D4-7E11-496E-8292-4ED5C0676C83

http://species-id.net/wiki/Epanerchodus_flagellifer

Figs 61 68 

#### Type material:

Holotype ♂ (NMNS-6561-001), Taiwan, Nantou County, Huisun timber land, 24.04.1998, leg. S.H. Wu. Paratypes: 1 ♂ (NMNS-6561-002), same locality, together with holotype; 1 ♂ (NMNS-6561-003), same locality, 04.1998; 1 ♂ (ZMUM), same locality, 25.04.1999; 1 ♂ (TFRI), same locality, 24.04.1999, all leg. S.H. Wu.

#### Name:

To emphasize the remarkably long, flagelliform distal part of the gonopod telopodite.

#### Diagnosis:

Differs from other *Epanerchodus* species in the distal half of the gonopod telopodite being particularly long, flagelliform, coupled with the presence of two rounded teeth at the base of the endomere (see also Key below). From the other congeners known from Taiwan, this new species differs also in the absence of sphaerotrichomes on ♂ legs.

#### Description:

**Figures 61–65.**
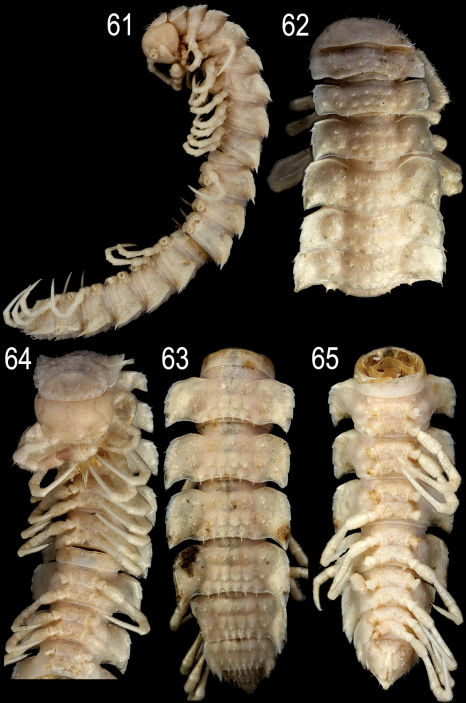
*Epanerchodus flagellifer* sp. n., ♂ paratype. **61** habitus, lateral view **62, 64** anterior portion of body, dorsal and ventral views, respectively **63, 65** posterior portion of body, dorsal and ventral views, respectively. Photographed not to scale.

Length of both sexes ca 8–11 mm; width of pro- and metazona varying between specimens from 0.8–1.0 to 1.4–1.5 mm, respectively. Holotype ca 9 mm long, and 0.9 and 1.4 mm wide on pro- and metazona, respectively. Coloration in alcohol pallid to light grey- to red-brown (Figs 61–65).

All characters as in *Epanerchodus orientalis* except as follows.

Antennae rather long and evidently clavate (Figs 61, 64), reaching behind segment 3 dorsally.

In width, collum < segment 2 < head = 3 = 4 < 5–15(16) (Fig. 62), thereafter body gradually tapering towards telson (Fig. 63). Paraterga well-developed, starting from collum, set high, subhorizontal to very faintly upturned, mostly nearly level to a very faintly convex dorsum, only on collum and segment 2 lying clearly below dorsum; paraterga on collum small, subtriangular, a small lateral incision in front of a rounded caudal corner; front shoulders drawn forward and slightly convex only paraterga 2 and 3, straight on paraterga 4, thereafter increasingly well rounded and directed increasingly caudolaterad; caudal edge straight on segments 2–4, thereafter increasingly bisinuate and acutangular caudolaterally; starting from segment 10, caudal corners extending increasingly beyond rear tergal contour, spiniform on segments 17–19 (Figs 61–65). All poreless segments with three, all pore-bearing ones with four, small but evident incisions, each usually bearing a small seta on top at lateral margin. Metatergal sculpture typical, moderately developed, with three indistinct transverse rows of setiferous, polygonal bosses (Figs 62, 63). Tergal setae very short, mostly retained, a little longer only on collum and in rear row on metatergum 19. Stricture between pro- and metazona wide and smooth. Limbus very thin, microdenticulate. Epiproct rather short, conical (Fig. 65), only slightly bent ventrad, preapical papillae evident. Hypoproct semi-circular (Fig. 65); caudal, paramedian, setiferous papillae evident and well-separated.

**Figures 66–68.**
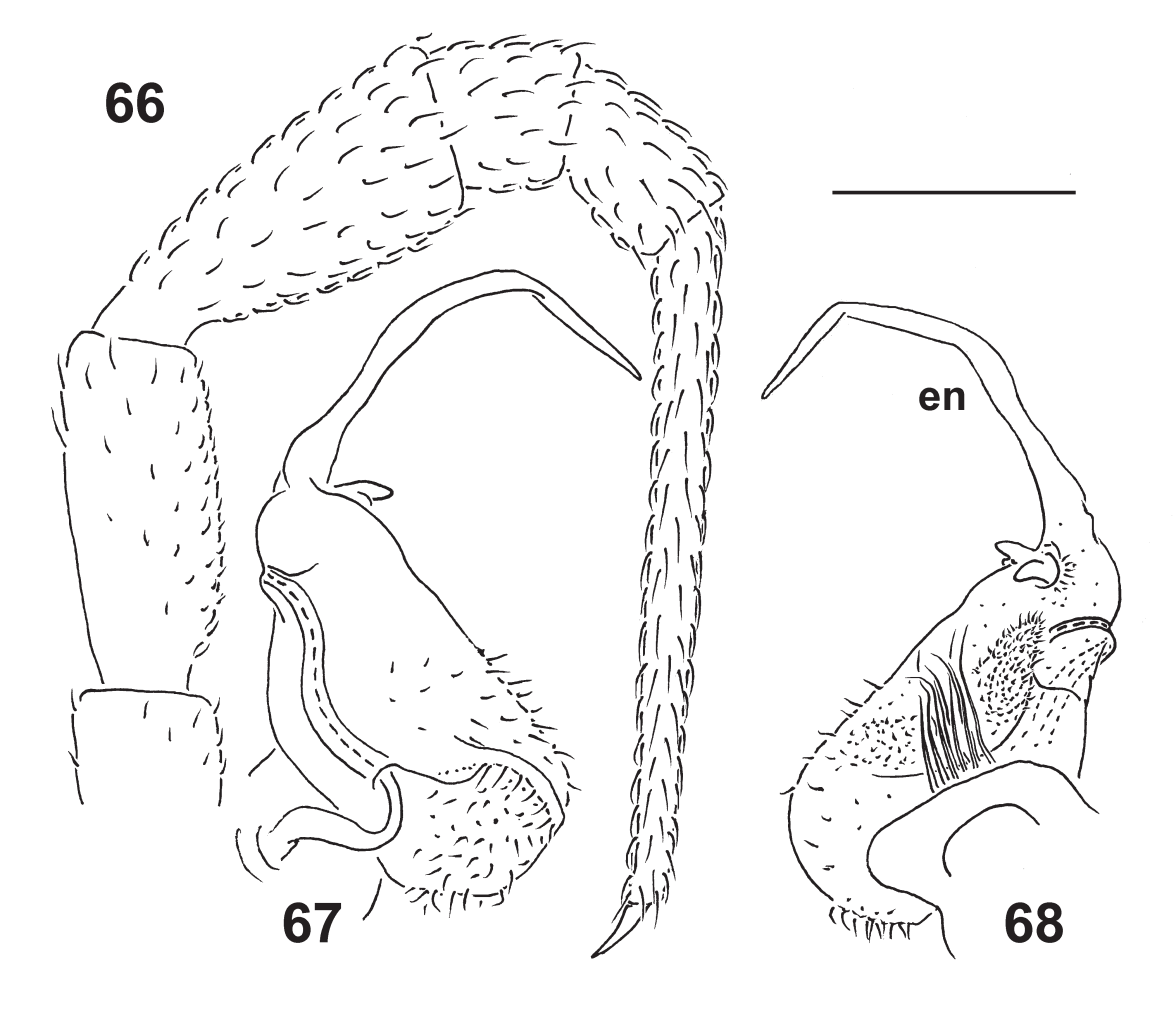
*Epanerchodus flagellifer* sp. n., ♂ paratype. **66** leg 9 **67, 68** left gonopod, mesal and lateral views, respectively. Scale bar: 0.2 mm.

Sterna without modifications, very densely setose. Legs rather long, slender, only slightly incrassate (Figs 61, 64–66), ca 1.7–1.8 times (♀) as long as midbody height, prefemora not swollen dorsally, sphaerotrichomes missing (Fig. 66).

Gonopods (Figs 67, 68) with large, subquadrate, medially fused coxae carrying only a few long setae ventrally. Telopodite simple and especially slender, prefemoral portion relatively short, endomere (**en**) flagelliform, with two small dentiform outgrowths at base; hairy pulvillus evident.

#### Remarks.

This small species is remarkable in showing a particularly long, flagelliform endomere, coupled with only two short outgrowths at its base.

In Taiwan, *Epanerchodus flagellifer* sp. n. occurs very locally, having been encountered only at a single locality (Map 2).

### Key to Epanerchodus species known from Taiwan (valid mostly for adult males)

**Table:** 

1	Gonopod coxae unusually densely setose, while telopodite particularly stout: prefemoral, setose part at least half as long as entire telopodite (Figs 43, 44, 59, 60)	2
–	Gonopod coxae usual, with only a few strong setae, while telopodite elongate: prefemoral, setose part less than half as long as entire telopodite (Figs 32, 52, 53, 67, 68)	3
2	♂ legs devoid of sphaerotrichomes (Fig. 66). Gonopod endomere (en) like a short spine (Figs 67, 68)	*Epanerchodus curtigonopus* sp. n.
–	all ♂ telopoditomeres with sphaerotrichomes (Fig. 42). Gonopod endomere (en) like a strong tooth (Figs 40, 43, 44)	*Epanerchodus pinguis* sp. n.
3	Body usually larger, 11–19 mm long. Gonopod telopodite highly variable, complex: exomere (ex), if present, rudimentary, while endomere (en) branching (Figs 29, 30, 32–36)	*Epanerchodus orientalis*
–	Body usually smaller, 8–11 mm long. Gonopod telopodite simple: exomere (ex), if present, strong and spiniform, while endomere (en) like a strong spine or a long flagellum	4
4	Gonopod telopodite biramous, exomere (ex) strong and spiniform, while endomere (en) like a strong spine ending in a strong uncus (Figs 52, 53)	*Epanerchodus bispinosus* sp. n.
–	Gonopod telopodite uniramous, exomere absent, endomere (en) flagelliform (Figs 67, 68)	*Epanerchodus flagellifer* sp. n.

## Conclusion

At present we can state that Taiwan supports a reasonably rich and quite peculiar polydesmid fauna represented by seven species in two genera. Of these, six species seem to be endemic, being largely restricted to the mountains in the central and northern parts of the island. Considering the distributions demonstrated by the Taiwanese Polydesmidae (Maps 1 and 2), allopatry is prevailing while sympatry, probably even syntopy, has only been observed in two places. Thus, the normally much larger *Nipponesmus shirinensis*, the slightly smaller *Epanerchodus pinguis* sp. n. and the smallest *Epanerchodus flagellifer* sp. n. co-occur at Huisun. Similarly, *Nipponesmus shirinensis*, *Nipponesmus minor* sp. n., *Epanerchodus orientalis* and *Epanerchodus bispinosus* sp. n., which also form a comparable succession of size decrease, are sympatric if not syntopic at Mei-Feng. Such examples seem to be best explained in terms of local niche segregation long documented elsewhere for a number of insular groups of Diplopoda (e.g. Enghoff 1983, Enghoff and Báez 1993, Golovatch and Enghoff 2003), including those of Taiwan (Golovatch et al. 2010).

The only polydesmid that is remarkably widespread and polymorphous in Taiwan, *Epanerchodus orientalis*, appears to be confined to lowland to foothill habitats (Map 2). For this reason alone, this species, which is also known to be extremely widely distributed, variable and lowland-dwelling across at least most of Japan, seems to be the only allochthonous element in the fauna of Polydesmidae of Taiwan, likely a later colonizer from Japan.

## Supplementary Material

XML Treatment for Nipponesmus shirinensis

XML Treatment for Nipponesmus minor

XML Treatment for Epanerchodus orientalis

XML Treatment for Epanerchodus pinguis

XML Treatment for Epanerchodus bispinosus

XML Treatment for Epanerchodus curtigonopus

XML Treatment for Epanerchodus flagellifer
